# Rosiglitazone drives cavin-2/SDPR expression in adipocytes in a CEBPα-dependent manner

**DOI:** 10.1371/journal.pone.0173412

**Published:** 2017-03-09

**Authors:** Björn Hansson, Catarina Rippe, Dorota Kotowska, Sebastian Wasserstrom, Johanna Säll, Olga Göransson, Karl Swärd, Karin G. Stenkula

**Affiliations:** Department of Experimental Medical Science, Lund University, Lund, Sweden; Tohoku University, JAPAN

## Abstract

Caveolae are abundant adipocyte surface domains involved in insulin signaling, membrane trafficking and lipid homeostasis. Transcriptional control mechanisms for caveolins and cavins, the building blocks of caveolae, are thus arguably important for adipocyte biology and studies in this area may give insight into insulin resistance and diabetes. Here we addressed the hypothesis that one of the less characterized caveolar components, cavin-2 (SDPR), is controlled by CCAAT/Enhancer Binding Protein (CEBPα) and Peroxisome Proliferator-Activated Receptor Gamma (PPARG). Using human mRNA expression data we found that SDPR correlated with PPARG in several tissues. This was also observed during differentiation of 3T3-L1 fibroblasts into adipocytes. Treatment of 3T3-L1-derived adipocytes with the PPARγ-activator Rosiglitazone increased SDPR and CEBPα expression at both the mRNA and protein levels. Silencing of CEBPα antagonized these effects. Further, adenoviral expression of PPARγ/CEBPα or Rosiglitazone-treatment increased SDPR expression in primary rat adipocytes. The myocardin family coactivator MKL1 was recently shown to regulate SDPR expression in human coronary artery smooth muscle cells. However, we found that actin depolymerization, known to inhibit MKL1 and MKL2, was without effect on SDPR mRNA levels in adipocytes, even though overexpression of MKL1 and MKL2 had the capacity to increase caveolins and cavins and to repress PPARγ/CEBPα. Altogether, this work demonstrates that CEBPα expression and PPARγ-activity promote SDPR transcription and further supports the emerging notion that PPARγ/CEBPα and MKL1/MKL2 are antagonistic in adipocytes.

## Introduction

Adipocytes constitute a major energy store and altered adipose tissue function is associated with development of obesity and diabetes. The plasma membrane of adipocytes is endowed with bulb-shaped invaginations called caveolae, enriched in cholesterol and sphingolipids [1]. These organelles are involved in insulin signaling, membrane trafficking and lipid homeostasis [2]. The integral membrane protein caveolin exists in three isoforms (CAV1-3), and is required for caveola formation [3]. In recent years, it has been demonstrated that the cavin proteins (PTRF, SDPR, PRKCDBP, MURC) associate with the cytosolic side of caveolae and assist in their maturation [4, 5]. In humans, mutations in CAV1, CAV3 and PTRF cause lipo- and muscular dystrophies [6–8]. Moreover, genetic ablation of caveolae in mice causes impaired insulin receptor substrate (IRS)-1 signaling and insulin resistance [9–12]. Exploring the transcriptional regulation of caveolar components is therefore central for understanding adipocyte biology and may shed light on the link between caveolae and diabetes.

One of the less well studied cavins is cavin-2 (SDPR). This is a phosphatidylserine-binding protein [13] that was originally considered to target protein kinase C to caveolae [14]. In a landmark paper by Hansen et al. [15] it was found that cavin-2 knockdown led to a reduced abundance of caveolae at the surface of HeLa cells, whereas overexpression led to membrane tubulation. On the basis of these findings, cavin-2 was proposed to be critical for membrane bending in caveolae. Work on adipocytes, where cavin-2 is highly expressed [16], demonstrated that cavin-2 is an integral component of caveolae [16, 17]. Accordingly, cholesterol depletion led to degradation of cavin-2, redistribution of cavin-1 (PTRF), and loss of adipocyte caveolae [18]. Cavin-2 is also highly expressed in endothelial cells and *in vivo* knockout of cavin-2 reduces the density of caveolae in lung endothelial cells, but not in heart endothelial cells [19]. Cavin-2 therefore appears to be required for caveolae in adipocytes and lung endothelial cells, but possibly not for caveolae in other cell types. Such differential dependence on cavin-2 is likely dictated by the absolute expression levels, and by the cavin-2 to cavin-3 ratio, as the latter two cavins compete for binding to cavin-1 in a heterotrimeric complex [5]. Therefore, to understand how caveolae are generated in adipocytes and endothelial cells we must understand how cavin-2 expression is regulated.

Some recent inroads into the transcriptional control of cavin-2 have been made. Regazzetti et al. [20] demonstrated that hypoxia reduces cavin-2 expression via hypoxia inducible factors. Cavin-2 was also shown by us to be regulated by actin polymerization via the myocardin family coactivator MKL1 (MRTF-A) [21]. MKL1 is, to the best of our knowledge, the only positively acting transcriptional control mechanism for cavin-2 that has been described. Because recent studies have demonstrated that MKL1 counteracts adipogenesis, and because MKL1 is dramatically repressed in this process [22, 23], it is difficult to imagine that MKL1 could be responsible for the high expression of cavin-2 in adipocytes. Some other, positively acting, transcriptional control mechanism for cavin-2 is therefore sought to explain its high expression in adipose tissue [16].

Adipocyte formation, or adipogenesis, is a complex process involving many different transcription factors. The process can be modeled *in vitro* using e.g. 3T3-L1 cells exposed to adipogenic cues [24]. Two critical transcription factors are CEBPα (CEBPA) and PPARγ (PPARG) [25]. These transcription factors induce each other’s expression and jointly target key genes in adipogenesis [26]. Here we explored the possible role of CEBPα and PPARγ for transcriptional regulation of cavin-2 in primary adipocytes. We demonstrate that PPARγ and cavin-2 correlate in human tissues, that cavin-2 is upregulated during adipocyte differentiation in parallel with CEBPα and PPARγ induction, and that CEBPα expression and PPARγ-activation induces cavin-2 in primary adipocytes and their precursors. In all, these findings suggest that CEBPα/PPARγ may represent an important transcriptional control mechanism for cavin-2/SDPR, and thus for caveolae, in adipocytes.

## Materials and methods

### Material

3T3-L1 cells were purchased from American Type Culture Collection. Quantifast SYBR Green RT-PCR kit (Qiagen, Holden, Germany), adenoviral vectors for GFP, MKL1, MKL2, CEBPα and PPARγ (GenBank accession number CU013410, BC171750, BC028890, and BC006811), were produced by Vector BioLabs Inc, (Philadelphia, USA). The PPRE-x3-TK-Luc reporter plasmid was a kind gift from Bruce Spiegelman (Addgene plasmid #1015) and SDPR reporter plasmid was obtained from Acive Motif (Nysdam, Belgium). Heat shock protein (HSP)90 and anti-caveolin-1 and -2 antibodies were from Cell Signaling Technologies (Danvers, USA). The anti-perilipin antibody was from Vala Science (San Diego, USA). Antibodies against PTRF (cavin-1) (ab48824) and SDPR (cavin-2) (AF5759) were from R&D Systems (Minneapolis, USA) whereas the PRKCDBP (cavin-3) antibody was from Proteintech (Rosemont, USA) (16250-1-AP).

### Ethics statements

The Malmö/Lund experimental animal ethics Committee (Lund, Sweden) approved all procedures.

### Preparation of primary adipose cells and adenoviral expression

Rat epididymal adipose cells were isolated from male Sprague Dawley rats, as described previously [27]. Cells were suspended in DMEM with Gentamicin (100 μg/ml), phenyl-isopropyl-adenosin (PIA) (200 nM) and albumin (3.5% w/v), and cultured at 5% CO_2_, 37°C for 20 hours with eGFP-MKL1, MKL2, CEBPα, PPARγ1 or control (GFP) virus. Virus treatment caused between 61 to 460-fold overexpression (S1 Fig, panel A). For MKL1, which had an eGFP tag, we confirmed overexpression by fluorescence imaging, showing that eGFP-MKL1 localized in the cytoplasmic rim, and prominently in the nucleus (S1 Fig, panel B).

### RNA isolation

Cells and intact tissue were lysed and homogenized in Qiazol™ lysis reagent and RNA was isolated using RNeasy® Mini Kit (Qiagen) according to the manufacturer’s recommendations.

### RT-qPCR

PCR-reactions were performed using the Quantifast SYBR Green RT-PCR kit (Qiagen #204156) and Quantitect primer assays for 18S, MKL1, MKL2, CAV1, CAV2, PTRF, SDPR, PRKCDBP, PPARγ1, and CEBPα. Primer sequences are considered proprietary information by Qiagen. mRNA expression levels were measured using a StepOnePlus real-time thermal cycler (Applied Biosystems, Waltham, USA) and quantitated using the ΔΔC_T_ method as described by Livak and Schmittgen [28]. 18S mRNA expression levels were used for normalization throughout.

### Transfection and luciferase assay

Isolated adipocytes were electroporated as described previously [29]. Briefly, isolated adipose cells were suspended (40% v/v) in DMEM supplemented with Gentamicin (100 μg/ml) and PIA (200 nM) and electroporated (3×12 ms, 500 V/cm) (Harvard Apparatus, Holliston, USA) with plasmids as indicated (reporter:promoter at 10:1). Afterwards, the cells were transferred into DMEM with Gentamicin, PIA, and BSA (3.5% w/v) and cultured for 20 h at 37°C in 5% CO_2_. Cells were lysed in an equal amount of Promega passive lysis buffer and centrifuged at 1000xg for 10 min at 4°C. Luciferase activity was measured in a Glomax luminometer (Promega) using the Dual Luciferase Reporter (Promega) (PPRE) or LightSwitch Luciferase (ActiveMotif) (SDPR) systems.

### Cell culture and CEBPA silencing

3T3-L1 fibroblasts were cultured and differentiated as previously described [30]. In short, cells were cultured at sub-confluence in DMEM medium containing 10% FCS (v/v) and 1% penicillin/streptomycin (v/v) in an atmosphere with 5% CO_2_ at 37°C. For differentiation, cells were incubated with DMEM medium containing 10% FCS (v/v), 1% penicillin/ streptomycin (v/v), 0.5 mM 3-isobutyl-1-methylxanthine (IBMX), 10 μg/ml insulin and 1 μM dexamethasone for 48 h two days post confluence (designated day 0). Medium was changed to DMEM with 10 μg/ml insulin for 48 h and thereafter cells were cultured in DMEM containing 10% FCS (v/v) and 1% penicillin/ streptomycin (v/v) until day 9. In a subset of experiments, 3T3-L1 cells were treated with Rosiglitazone (1 μM, Sigma) for the time indicated in the figures. CEBPA silencing was performed as previously described [31] by electroporation at day 4, using siRNA targeting CEBPA (Ambion, ID: 63853 and 63854) or scrambled (Scr) siRNA (Ambion, Neg control #1 siRNA). After 24 hours, cells were stimulated for 20 hours with Rosiglitazone before collecting RNA and protein as described above.

### Oil Red O staining

3T3-L1 cells (day -2 and 9) were washed in PBS, fixed with 4% formaldehyde for 1h and washed twice with water. Cells were incubated with Oil Red O (Sigma) solution (8.57mM Oil Red O in isopropanol) mixed 6:4 with water for 1 h and washed twice in water. Representative images were captured using an Olympus 1X71 system (Center Valley, USA), 10x objective NA 0.25.

### Confocal imaging

Preparation of cells for microscopy was done as previously described [30]. Briefly, isolated primary adipocytes were washed twice in Krebs-Ringer (KRH) buffer containing 25 mM Hepes pH 7.4, 200 nM adenosine, 2 mM glucose, followed by fixation in 4% paraformaldehyde (PFA) for 6 min, and then washed twice in PBS pH 7.4, followed by blocking and permeabilization in KRH buffer with 0.1% Saponin for 30 min. To visualize polymerized actin, fixed cells were incubated with fluorescent conjugated Phalloidin-647 (Molecular Probes, Thermo Fisher Scientific, Waltham, USA) at a concentration of 165 nM for 20 minutes at room temperature. Cells were washed twice in PBS and imaged using a Nikon A1 plus confocal microscope with a 60x Apo DIC oil immersion objective, NA 1.40 (Nikon Instruments Inc.). Phalloidin-647 was excited at 640 nm and emission detected between 663–738 nm. All images were identically subjected to background subtraction and threshold settings. Images were acquired with NIS-elements, version: 4.50.02, (Laboratory Imaging).

### Western blotting

Western blotting was performed as described previously [30]. In short, following incubations, cells were washed with KRH medium without BSA and subsequently lysed in a buffer containing 50 mM Tris/HCl pH 7.5, 1 mM EGTA, 1 mM EDTA, 0.27 M sucrose, 1% NP-40, and complete protease-and phosphatase inhibitor cocktail (Roche, Basel, Switzerland) (one tablet each/10 ml buffer). Lysates were centrifuged for 10 min at 13000xg and protein concentrations were determined using the Bradford method. Samples were subjected to polyacrylamide gel electrophoresis and electro-transfer to nitrocellulose membranes. Membranes were blocked and probed with the indicated antibodies. Detection was performed using horseradish peroxidase conjugated secondary antibodies and enhanced chemiluminescence reagent. The signal was visualized using a BioRad Image camera (Biorad, Hercules, USA).

## Results

### PPARγ expression correlates with expression of cavin-2/SDPR in human tissues

**Fig 1:**
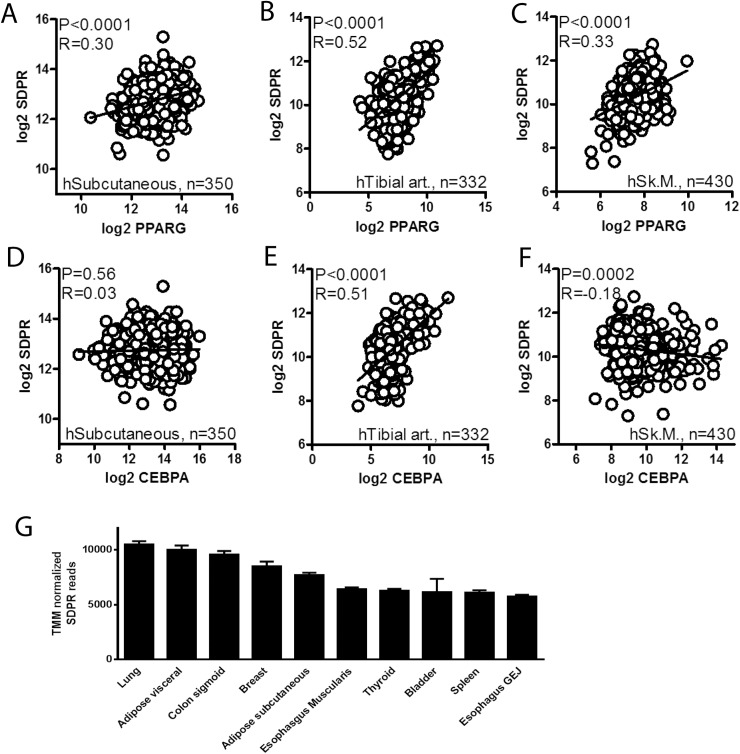
PPARγ expression correlates with cavin-2/SDPR expression in human tissues. Panels **A** through **F** show correlations between PPARG and SDPR and between CEBPA and SDPR mRNA levels in the indicated human tissues. RNA-Seq data is from the Genotype-Tissue Expression (GTEX) portal [32] and normalization was according to the trimmed mean of M-values method. Correlations were tested using the Spearman method in GraphPad Prism 5. Nominal P-values (P) and Spearman's rank correlation coefficients (R) are given in the panels. Panel **G** shows TMM normalized RNA-Seq reads (means ± SEM) for SDPR in the top ten expressing tissues in the database.

We approached the question of transcriptional control mechanisms for cavin-2 (SDPR) using mRNA expression data obtained from the Genotype-Tissue Expression (GTEx) portal [32]. Correlations at the mRNA levels for SDPR versus PPARγ (PPARG) and for SDPR versus CEBPα (CEBPA) were examined using Spearman statistics. Significant positive correlations between PPARG and SDPR were observed in human subcutaneous adipose tissue, in tibial artery, and in skeletal muscle (Fig 1A–1C). For CEBPA, on the other hand, no correlation was seen in adipose tissue, whereas a strong positive correlation was present in the tibial artery, and a weak negative correlation was observed in skeletal muscle (Fig 1D–1F). We also found a positive correlation between CEBPG and SDPR in adipose tissue (P<0.0001, R = 0.21, not shown). These findings suggested the possibility that PPARγ may contribute to the transcriptional control of SDPR. Among the five human tissues with the highest SDPR expression (TMM normalized reads), at least two, possibly three including breast, are dominated by adipocytes (Fig 1G). We therefore henceforth focused on regulation of SDPR in adipocytes and their precursors.

### PPARG and CEBPA correlate with cavin-2 expression during adipocyte differentiation

**Fig 2:**
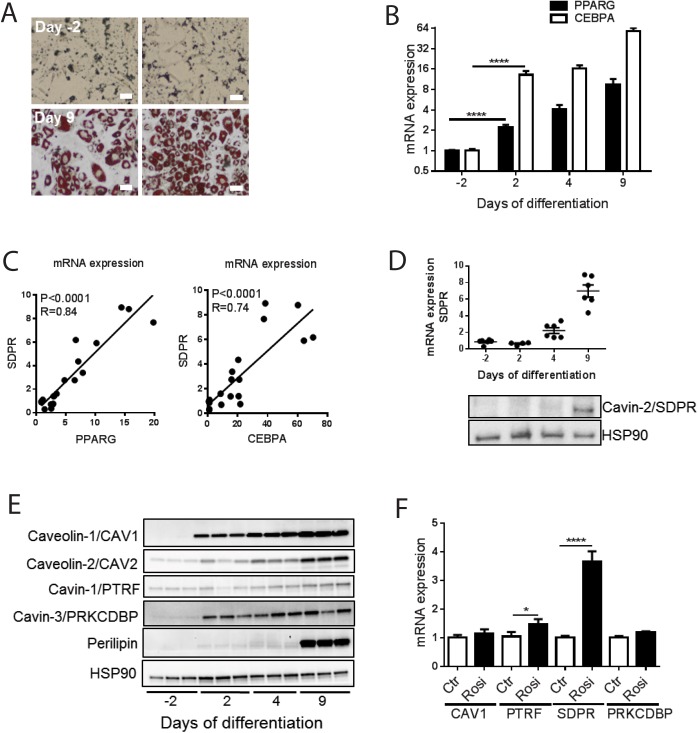
PPARG, CEBPA and SDPR expression during adipocyte differentiation of 3T3-L1 cells and the effect of the PPARγ activation using rosiglitazone. 3T3-L1 cells were treated with adipogenic cocktail on day 0 and allowed to differentiate into adipocytes for 9 days. Panel **A** shows two representative light microscopy images of 3T3-L1 cells incubated with Oil-Red O prior to differentiation (day -2), and following 9 days of differentiation. Bar = 10 μm. Panel **B** shows mRNA expression for PPARG and CEBPA, measured by qPCR at different time points of differentiation (-2, 2, 4 and 9 days). Panel **C** shows correlations between PPARG and SDPR and between CEBPA and SDPR, respectively, during adipocyte differentiation. Panel **D** shows mRNA expression and western blots for SDPR, and panel **E** western blots for caveolins and cavin-1 and -3 at different time points of differentiation. Cell lysates were collected and subjected to western blotting to detect total protein levels caveolin-1 (CAV1), caveolin-2 (CAV2), cavin-1 (PTRF), cavin-3 (PRKCDBP) and perilipin. HSP90 was used as loading control. Representative blots from n = 3 independent experiments with each sample run in triplicates are shown. Panel **F** shows the effect of treatment with the PPARγ activator rosiglitazone (1 γM, from day 4 to day 9) on the mRNA levels of caveolins and cavins. DMSO was used as vehicle control. Data in (**B** and **F**) is presented as means±SEM, *p≤0.05, **p≤0.01, and ****p≤0.0001.

Differentiation of 3T3-L1 cells into adipocytes in culture was initiated by adding an adipogenic cocktail. Conversion into adipocytes was examined using Oil-Red O staining, confirming that a majority (80%) of the cells acquired lipid droplets 9 days after commencement of differentiation compared with non-differentiated (-2 days) cells (Fig 2A). mRNA was extracted at day -2, 2, 4 and 9 for qPCR analyses. The expression of PPARG and CEBPA increased gradually during differentiation as expected (Fig 2B). Both PPARG and CEBPA were found to correlate with the expression of SDPR, and, in accordance with our findings in human adipose tissue, correlation with PPARG was tighter (Fig 2C, left vs. right). The expression of SDPR mRNA increased exponentially and in parallel with the protein during differentiation (Fig 2D). Other caveolae proteins along with perilipin, a definitive adipocyte marker, increased as well (Fig 2E).

3T3-L1 cells were next treated with the PPARγ activator rosiglitazone (Rosi, 1 γM) during differentiation (from day 4 through day 9). As predicted from the correlation analyses, Rosiglitazone significantly increased the expression of SDPR (Fig 2F). A small effect was seen on cavin-1 (PTRF), but levels of caveolin-1 (CAV1) and cavin-3 (PRKCDBP) were unaffected (Fig 2F). Our findings so far therefore suggested that cavin-2 may be regulated by PPARγ/CEBPα during adipocyte differentiation.

### Overexpression of CEBPα and PPARγ activation increase SDPR expression in primary adipocytes

**Fig 3:**
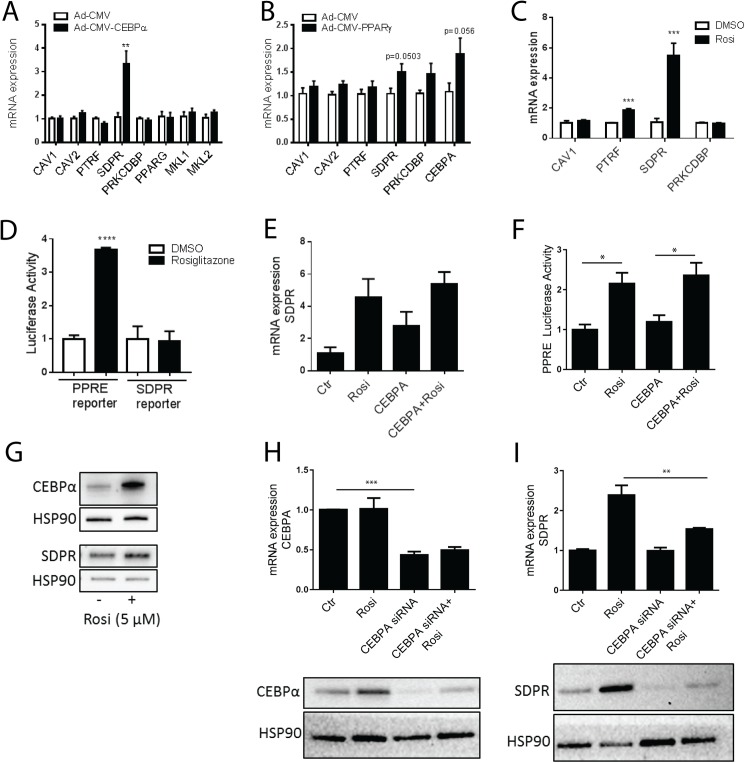
Overexpression of CEBPα and PPARγ activation increase SDPR expression in primary adipocytes. Panels **A** and **B** show qPCR analysis of caveolin and cavin expression in primary rat adipocytes following 20–24 h of incubation with adenovirus encoding CEBPα (Ad-CMV-CEBPα) (**A**), PPARγ (Ad-CMV-PPARγ) (**B**) or control adenovirus (Ad-CMV-null). n = 6 independent experiments. Adipocytes were incubated with Rosiglitazone (5 μM) or vehicle (DMSO) for 20–24 h, and the relative expression of CAV1, CAV2, PTRF, SDPR, and PRKCDBP analyzed using qPCR (**C**, n = 4 independent experiments). In panel **D** (left bars), adipocytes were transfected with PPRE luciferase reporter and Renilla reporter plasmids, followed by overnight (20 h) incubation with Rosiglitazone (5 μM) or vehicle (DMSO). Luciferase activity was measured in cell lysates using a luminometer, and the values normalized to Renilla luciferase activity. Each sample was measured in triplicate (n = 3 independent experiments). Adipocytes were also transfected with an SDPR promotor reporter (right bars) and incubated overnight (20 h) with Rosiglitazone (5 μM) or vehicle (DMSO). Cell lysates were subjected to luciferase activity assay and each sample was measured in triplicate. n = 3 independent experiments. Adipocytes were incubated with DMSO (ctr), CEBPα adenovirus, Rosiglitazone (5 μM), or their combination overnight (20 h), and analyzed using qPCR (**E**, n = 3 independent experiments). In panel **F**, adipocytes were transfected with PPRE luciferase reporter and Renilla reporter plasmids, followed by overnight (20 h) incubation with adenovirus encoding CEBPα, Rosiglitazone (5 μM) or vehicle (DMSO) as indicated. Cell lysates were subjected to luciferase activity assay and each sample was measured in duplicate. n = 3 independent experiments. Western blot showing CEBPα and SDPR in cell lysates from primary adipocytes incubated with or without Rosiglitazone 20 h (DMSO, ctr). HSP90 was used as loading control. (**G**). In **H**, CEBPA was silenced using siRNA (or scrambled (ctr)) in 3T3-L1 cells on day 4 of differentiation. At day 6, cells were stimulated with Rosiglitazone (1 μM) for 20 h. n = 3 independent experiments, each sample run in duplicate. CEBPα silencing was confirmed by western blotting, representative image is shown below graph (**H**). mRNA expression of SDPR following CEBPA silencing is shown in **I**. Protein level of SDPR shown below graph (**I**). HSP90 used as loading control (**H** and **I**). Data is presented as means±SEM, *p≤0.05, **p≤0.01, ***p≤0.001, and ****p≤0.0001.

We next used adenoviral overexpression to examine if CEBPα and PPARγ can drive expression of cavin-2 in primary white adipocytes from rat. Overexpression of CEBPα caused a 3-4-fold increase of SDPR expression, whereas other cavins and caveolins analyzed were unaffected (Fig 3A). PPARγ and the myocardin family coactivators MKL1 and MKL2 were similarly unchanged (Fig 3A). Overexpression of PPARγ induced expression of SDPR (Fig 3B), albeit with borderline significance (p = 0.0503 using a two-sided t-test, p = 0.02 using a one-sided t-test). Since PPARγ is a ligand-activated receptor, we also treated primary adipocytes with rosiglitazone (5 μM, 20 h). qPCR analysis demonstrated a marked, 5-6-fold, induction of SDPR in Rosiglitazone-treated adipocytes (Fig 3C). PTRF also increased, but the expression of CAV1 and PRKCDBP was unaffected (Fig 3C).

PPARγ activity and SDPR promoter activation were next examined using luciferase reporter assays. These assays showed that Rosiglitazone caused a 3-fold increase in PPARγ activity compared with DMSO-treated control cells (Fig 3D), providing a positive control for the treatment. The proximal SDPR promoter (≈1000 nt) was however not activated by Rosiglitazone from the same batch and at the same concentration (Fig 3D), suggesting involvement of a distal enhancer.

We also examined if CEBPα and PPARγ were acting synergistically. Somewhat to our surprise, the effects of Rosiglitazone and CEBPα appeared to be non-additive (Fig 3E). The PPARγ response element (PPRE) reporter was not responsive to CEBPα expression, ruling out that CEBPα works through PPRE (Fig 3F), but leaving the possibility that Rosiglitazone acts indirectly on SDPR through PPARγ-mediated induction of CEBPα. This hypothesis was supported by the observation that overnight incubation with Rosiglitazone increased the protein levels of CEBPα and SDPR (Fig 3G). Silencing of CEBPA in 3T3-L1 cells (Fig 3H) significantly suppressed the Rosiglitazone-driven SDPR expression (Fig 3I), without altering the non-stimulated SDPR level (Fig 3I, Ctr versus CEBPA siRNA). Western blotting confirmed that Rosiglitazone increases both CEBPα (Fig 3H, lower panel) and cavin-2 (Fig 3I, lower panel) expression in 3T3-L1 cells, and that CEBPA silencing diminished this effect (Fig 3H and 3I, lower panels). These findings show that CEBPα and PPARγ, key lineage specifiers in adipocytes, have the ability to drive expression of SDPR in both mature adipocytes and their precursors and that the level of CEBPα, indirectly controlled by PPARγ, exerts a transcriptional drive on cavin-2.

### MKL1 and MKL2 drive expression of caveolins and cavins but SDPR expression is unresponsive to actin depolymerization in primary adipocytes

**Fig 4:**
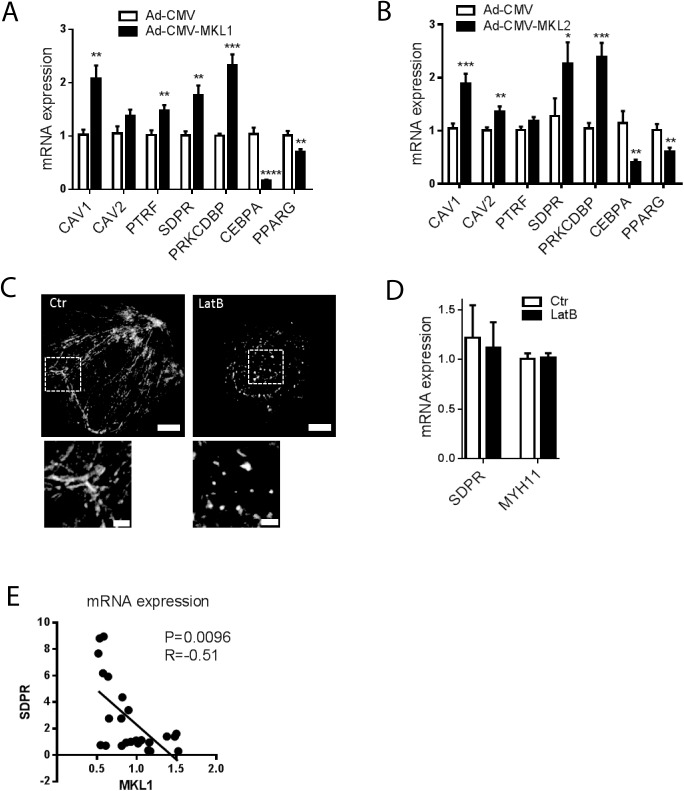
MKL1 and MKL2 drive expression of caveolins and cavins but SDPR expression is unresponsive to actin depolymerization in primary adipocytes. Panels **A** and **B** show expression of CAV1, CAV2, PTRF, SDPR and PRKCDBP in primary adipocytes following incubation with adenovirus encoding MKL1 (Ad-CMV-MKL1, **A**), MKL2 (Ad-CMV-MKL2, **B**) or control adenovirus (Ad-CMV-null). n = 6–10 independent experiments. Panel **C** shows actin staining in control adipocytes (Ctr, DMSO) and in adipocytes treated with latrunculin B (LatB) for 16h. Bars represent 10 μm in upper panels. Magnifications of actin cables, and puncta, respectively, are shown in the lower panels where bars represent 2 μm. n = 3 independent experiments where 10–15 images were captured per experiment. Panel **D** shows the SDPR mRNA level in primary adipocytes following incubation in the absence and presence of LatB (10 μM) for 16 h. Control cells (ctr) were incubated with DMSO. n = 3 independent experiments. Panel **E** shows that the mRNA levels for MKL1 and SDPR are negatively correlated during adipocyte differentiation. Data in panels **A** through **D** is presented as means±SEM, *p≤0.05, **p≤0.01, ***p≤0.001 and ****p≤0.0001.

In recent work it was found that myocardin family coactivators (MYOCD and MKL1) are able to regulate the expression of all caveolins and cavins in smooth muscle cells [21]. MKL1, which is highly expressed in mesenchymal cells, downregulates PPARγ and is repressed during early stages of adipogenesis [23]. This is critical for normal adipocyte maturation. In human adipose tissue, we found that MKL2 reads [32] were ten-fold higher than those of MKL1 (not shown). We therefore next explored the possible effects of both MKL1 and MKL2 on caveolin and cavin expression in adipocytes. By qPCR analysis, we found that viral overexpression of MKL1 and MKL2 increased the expression of essentially all caveolins and cavins in primary rat adipocytes and moreover repressed CEBPα and PPARγ (Fig 4A and 4B). Since the activities of MKL1 and MKL2 are inhibited by monomeric actin [33], we next treated adipocytes with the actin depolymerizing agent Latrunculin B (LatB, 10 μM). LatB disrupted actin filaments as shown using phalloidin staining and confocal microscopy (Fig 4C), providing a positive control for the treatment effect, but had no effect on SDPR mRNA expression (Fig 4D). The prototypical MKL1 target MYH11 was similarly unresponsive (Fig 4D). This is very different from smooth muscle cells where LatB reduces SDPR by ≈70% [21], arguing that SDPR expression is independent of actin polymerization, and thus presumably MKL1/MKL2 activity, in adipocytes.

Our experiments in adipocytes suggested the possibility that the transcriptional drive for SDPR may be shifted from MKLs towards PPARγ/CEBPα during adipogenesis. If this was the case, one might expect a negative correlation between MKL1 and SDPR during differentiation. This was indeed seen (Fig 4E), further supporting the notion that MKL1 and PPARγ/CEBPα are mutually antagonistic in the context of adipogenesis.

## Discussion

This is, to the best of our knowledge, the first report demonstrating that two regulators of adipocyte differentiation, CEBPα and PPARγ, regulate cavin-2 (SDPR) expression. Our current experiments constitute an important advancement of knowledge, showing significant induction of both cavin-1 (PTRF) and of cavin-2 (SDPR) following PPARγ activation. In addition, findings in the present work demonstrate that in primary adipocytes, CEBPα can control cavin-2 (SDPR) expression independently of PPARγ activity. Regulation of cavin-2 by CEBPα/PPARγ may be considered an explanation for the high expression of cavin-2 in adipose tissue ([16, 19], present study) and potentially also in endothelial cells, where PPARγ exerts important effects [34]. Loss of cavin-2 has been demonstrated to cause loss of caveolae in some tissues [15, 18, 19], supporting the view that it is an important organelle constituent. Our findings are therefore likely of direct relevance for biogenesis of caveolae.

Prior work has established that PPARγ may drive expression of CAV1 and CAV2 [35, 36]. One of those studies found that adenoviral transduction of PPARγ *in vivo* caused adipogenic transdifferentiation of hepatic cells together with a 30-fold induction of CAV1 [36]. The effect on CAV1 appears to be cell type-dependent, however, and in some cancer cell lines no effect of PPARγ activation on CAV1 was observed [35]. The basis of this variability remains unexplored and the finding that PPARγ regulates CAV1 predates the discovery of the cavins [37]. Here, we were unable to demonstrate an effect of PPARγ activation on the CAV1 mRNA level in primary adipocytes and 3T3-L1 cells during differentiation. A well-established paradigm in the caveolae field is that formation of caveolae provides a scaffold that protects caveolins and cavins from degradation. It is possible that the effect of PPARγ and CEBPα on cavin-2 may indirectly stabilize other caveolae constituents. This is also expected to provide an indirect way to regulate the density of caveolae at the cell membrane.

Beyond the novel finding that cavin-2/SDPR is controlled by CEBPα/PPARγ in adipocytes, our results also confirm and extend the recent demonstration that myocardin family coactivators control the expression of caveolins and cavins [21]. In our previous study [21] we found that myocardin and MKL1 control gene expression of cavolins and cavins in smooth muscle. Herein, we demonstrate that MKL2, similar to MKL1, has the ability to drive transcription of all caveolins and cavins except cavin-1 (PTRF), in terminally differentiated, primary adipocytes. Several lines of evidence however argue against the idea that MKL1 and/or MKL2 provide a transcriptional drive for SDPR in normal, healthy adipocytes. First, MKL1 and MKL2 have been demonstrated to be markedly reduced during adipogenesis [22], a process in which all caveolins and cavins increase. Here, we expand on the concept of a mutual antagonism between adipogenic and myogenic transcriptional influences by showing remarkable repression of both CEBPα and PPARγ following MKL transduction. Second, we demonstrate that depolymerization of actin using Latrunculin B, which inhibits MKL activity and represses cavin-2/SDPR by 70% in smooth muscle [21], is without effect on cavin-2/SDPR (and caveolin-1) in primary rat adipocytes. Because Latrunculin B had an effect on actin polymerization in adipocytes, and in view of our finding that MKL mRNA was detectable, our results suggest that the MKLs may be transcriptionally silent in adipocytes, perhaps due to a high G- to F-actin ratio. An exciting possibility is that this would allow MKLs to assume a role under pathological conditions, such as in adipocyte hypertrophy, often linked to obesity and insulin resistance.

At least two observations in the present study merit special consideration because they are seemingly contradictory. The first is our finding that correlations between CEBPα and SDPR in human tissues are less consistent than are correlations between PPARγ and SDPR, despite apparently larger effects of CEBPα than of PPARγ overexpression *in vitro*. We can only speculate on the basis of this discrepancy. One potential explanation is that PPARγ overexpression was made in mature adipocytes where the expression is already very high. PPARγ was thus potentially present at saturating concentrations, and could only be further activated via a ligand. Alternatively, the smaller effect on SDPR was caused by the relatively smaller degree of overexpression (S1 Fig, panel A). With regard to the poor correlation between CEBPα and SDPR at the tissue level, we speculate that presence of other cell types may represent a confounding factor, but other CEBP isoforms, including CEBPG, could also play a role. Another perplexing finding was that rosiglitazone failed to directly activate an SDPR promoter reporter, despite considerable effectiveness in the context of intact chromatin. One explanation could be that the promoter reporter encompassed only 1263 nucleotides upstream of the transcription start site and thus potentially did not include the relevant regulatory element(s). ChIP-Seq peaks at SDPR locus (UCSC Genome Browser) seem to support the presence of a distal enhancer, but we do not rule out other explanations. Indeed, PPARγ seems to be acting indirectly via induction of the CEBPα protein. This was suggested by the lack of additive effects of CEBPα overexpression and PPARγ activation, and the fact that the latter induced the CEBPα protein in two different adipocyte models. Knock down of CEBPα moreover partly mitigated the effect of Rosiglitazone on SDPR. Some effect of Rosiglitazone remained under these conditions, but this is to be expected given that CEBPα was only partly reduced (by 60%).

To summarize, novel findings in this study include the demonstration that PPARγ activation and CEBPα overexpression strongly regulate SDPR expression in adipocytes and that depolymerization of actin is without effects on SDPR expression in adipocytes, despite a capacity of MKL1 and MKL2 to increase mRNA levels of essentially all caveolins and cavins. This study, in conjunction with our previous work [21], thus highlights a considerable divergence between different cells types with regard to transcriptional control mechanisms of caveolar constituents. Such diversity may allow for tissue-specific pharmacological manipulation of caveolar function in diabetes and obesity.

## Supporting information

S1 Fig Efficiency of overexpression and MKL translocation. Panel **A** shows mRNA expression of CEBPA, MKL2 and PPARG in cells transduced with the respective virus. Panel **B** shows representative immunofluorescence images of a primary adipocyte expressing eGFP-MKL1 (nucleus in blue, left panel; eGFP signal in green, right panel), ScFigale bar = 10 μm.
(PDF)
