# Supplementary figures and images for: Rosiglitazone drives cavin-2/SDPR expression in adipocytes in a CEBPα-dependent manner
Source: PLoS One. 2017 Mar 9;12(3):e0173412. doi: 10.1371/journal.pone.0173412 (PMC5344386; doi:10.1371/journal.pone.0173412)

# S1 Fig

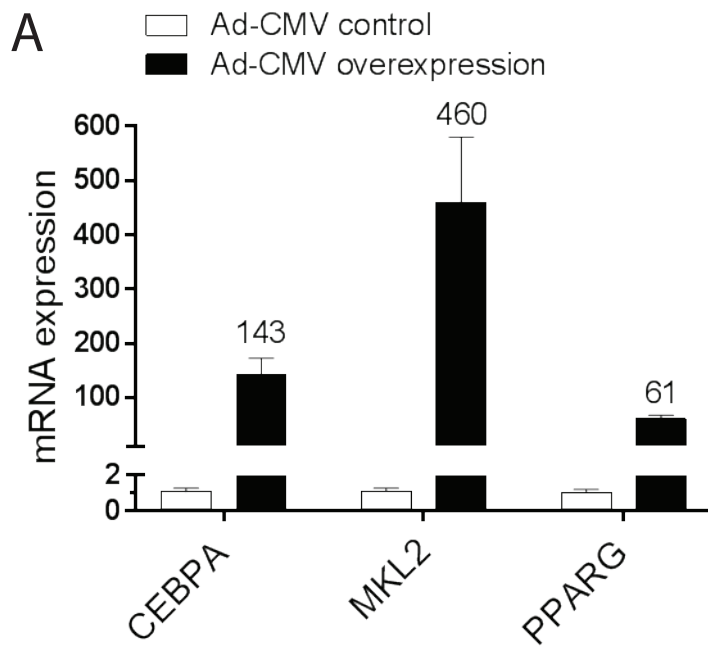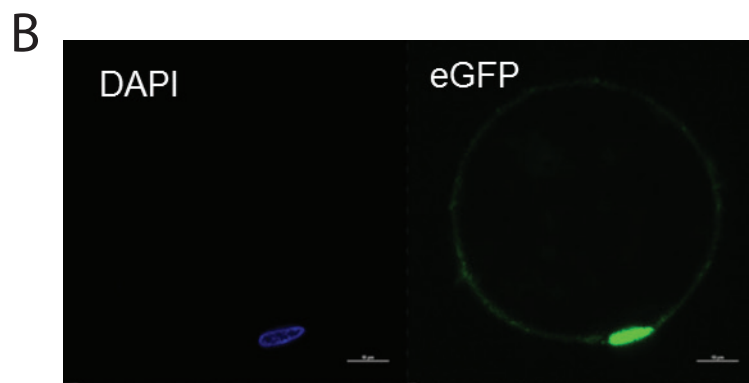

Supplement: S1 Fig — Panel A shows mRNA expression of CEBPA, MKL2 and PPARG in cells transduced with the respective virus. Panel B shows representative immunofluorescence images of a primary adipocyte expressing eGFP-MKL1 (nucleus in blue, left panel; eGFP signal in green, right panel), ScFigale bar = 10 μm. (PDF) [file pone.0173412.s001.pdf]
